# Cardiometabolic Risk and Cardiovascular Disease in Young Women With Uterine Fibroids

**DOI:** 10.7759/cureus.30740

**Published:** 2022-10-26

**Authors:** L M Brewster, Yentl Haan, Gert A van Montfrans

**Affiliations:** 1 Cardiovascular Disease, Creatine Kinase Foundation, Amsterdam, NLD; 2 Internal Medicine, Academic Medical Center (AMC), Amsterdam, NLD

**Keywords:** population based research, women's health, cardiovascular disease, diabetes mellitus, hypertension, uterine fibroids

## Abstract

Uterine fibroids are associated with hypertension and cardiometabolic risk factors, yet the onset and severity of cardiovascular disease (CVD) in women with fibroids remain understudied. We used data from the National Health and Nutrition Examination Survey to assess the association between uterine fibroids, cardiometabolic risk, and CVD (ischemic heart disease, heart failure, and stroke). Among 5,552 women aged 20-54 years in our sample. Hypertension was more common in those diagnosed with fibroids (n=700; 33.4% vs. 15.3% in controls, *p*<0.001), but these women were also older (44 (SD=7) years vs. 35 (SD=10) years). When stratified by median age, women aged 35 or younger with fibroids (n=97) were more likely than controls (n=2771) to have hypertension (14.1% vs. 2.4%), obesity (51.5% vs. 32.5%), and diabetes mellitus (4.8% vs. 1.9%). Women older than 35 with fibroids also had higher cardiometabolic risk and rates of CVD than controls (8.5% vs. 4.5%). The odds ratio of CVD in women with fibroids, compared to the controls, was 3.10 with 95% confidence interval (CI) of 2.21 to 4.34. The odds ratio decreased to 1.63 (95%CI: 1.11 to 2.38) after adjusting for age, BMI, African ancestry, fasting plasma glucose, cholesterol, education, smoking history, and postmenopausal status and to 1.60 (95%CI: 1.08 to 2.37) after further adjusting for systolic blood pressure. These data indicate that uterine fibroids are associated with a worse cardiovascular risk profile, including hypertension, in relatively young women, leading to early CVD. A women-specific research agenda is urgently needed to study the nature of these associations and design preventive strategies to prevent cardiovascular events in young women with fibroids.

## Introduction

Heart disease is a leading cause of mortality in women [1-3]. An estimated 275 million women worldwide have cardiovascular disease (CVD), and heart disease accounted for 35% of all deaths in women in 2019 [1-3]. General risk factors for CVD include lifestyle, hypertension, obesity, and diabetes [1-3]. Female-specific factors related to pregnancy, such as gestational hypertension and diabetes, are known to increase cardiovascular risk in later life [1,2,4]. Other non-pregnancy conditions in women also have been linked to cardiovascular risks, such as the presence of uterine fibroids, which is associated with increased blood pressure and other cardiovascular risk factors [5-8].

Uterine fibroids are a common condition in women [9]. Results of the National Health and Nutrition Examination Survey indicate an overall prevalence rate of 12% for symptomatic fibroids, which increases to 21% among women aged 50-54 years. However, cardiovascular endpoints in women with fibroids are not well studied [5-7]. Some studies report an increase in cardiovascular risk and coronary heart disease after hysterectomy, but these analyses included patients with a diversity of uterine disorders, many of whom also underwent oophorectomy, which might have affected cardiovascular endpoints [10-12]. Nevertheless, an important component of CVD prevention is the acknowledgement of female-specific risk factors across the life course that may warrant specialized approaches to risk assessment [1,2,4-7]. These risk factors include combined oestrogen-progesterone hormonal contraceptives, hypertensive disorders of pregnancy, preterm delivery, menopause, and menopausal hormone therapy [1,2,4], but the cardiovascular impact of fibroids is underrecognized and often not noted in papers or guidelines on women-specific management of cardiovascular risk [1,2,4]. Therefore, we assessed whether there is an independent association between uterine fibroids and cardiovascular events in a cross-sectional population setting.

In this paper, we report on the association between uterine fibroids, cardiometabolic risk, and CVD (ischemic heart disease, heart failure, and stroke) among 5,552 women aged 20-54 years. These data were previously presented at the 15th Oriental Congress of Cardiology in Shanghai in the Hypertension Forum, Session on Women and Hypertension Research, on May 28, 2021.

## Materials and methods

The National Center for Health Statistics conducts the National Health and Nutrition Examination Survey (NHANES), a nationally representative, multistage, stratified probability sample survey of the resident civilian noninstitutionalized United States (US) population. The survey comprises a face-to-face interview at home, as well as a physical examination conducted in a specially equipped mobile examination center, including the collection of blood and urine samples. The overall response rates range from 74% to 86% for the interview and 71% to 81% for the examination. Since 1999, NHANES data have been released every two years. We used data from four two-year cycles (1999-2006) for a representative sample of women aged 20-54 years. Of the initial sample of 6,508 women, 5,552 answered the question on fibroids. The primary outcome of the analysis was the odds ratio of CVD in women with or without fibroids.

The NHANES interview contains questions on demographics, reproductive health, medical conditions, and cardiovascular risk factors. In particular, women are asked whether a doctor or other health professional ever told them they had uterine fibroids, defined as benign (non-cancerous) tumors in various locations on or within the uterus. The NHANES physical examinations collect blood pressure (up to four measurements obtained by a trained examiner using a mercury sphygmomanometer to determine the mean of the two consecutive measurements with the smallest difference), weight (weighed on a digital scale while wearing a standard examination gown consisting of a disposable shirt, pants, and slippers), and standing height (measured using a stadiometer to the nearest 0.1 cm) data. Laboratory data include fasting plasma creatinine, lipid spectrum, and glucose. Spot urine specimens also are collected for measurement of albumin and creatinine levels.

Ancestry is self-reported and classified as Hispanic, White-European, African, or other. Education level is determined by the highest self-reported level of education and dichotomized into high school graduate or not. Smoking is categorized as current smoker, past smoker, or never smoked. Contraceptive hormones include oral use, skin patches, injections, and implants. To determine postmenopausal status, an algorithm based on the duration of amenorrhea, previous bilateral oophorectomy, and age is used [13]. Specifically, women with follicle-stimulating hormone levels ≥33 IU/L are considered postmenopausal [14]. Obesity is defined as a BMI≥30.0 kg/m2.

Hypertension is defined as systolic blood pressure ≥140 mmHg and diastolic blood pressure ≥90 mmHg or receiving antihypertensive drugs. Diabetes mellitus is defined as a fasting plasma glucose ≥7.0 mmol/L or the use of glucose-lowering medication. A previous diagnosis of hypertension or diabetes mellitus by a health professional also is recorded. Hypercholesterolemia is indicated if total cholesterol ≥6.2 mmol/L or the participant receives cholesterol-lowering medication.

We used the Chronic Kidney Disease-Epidemiology Collaboration (CKD-EPI) equation to calculate the estimated glomerular filtration rate (eGFR) [15]. Microalbuminuria was defined as an albumin-to-creatinine ratio ≥3.0 mg/mmol on spot urine. CKD was defined as eGFR<60 mL/min/1.73m2 or the presence of microalbuminuria. Cardiovascular risk categories (low to moderate, high, or very high) were based on the recommendation of the 2018 European Society of Cardiology/European Society of Hypertension guidelines for the management of arterial hypertension [16]. Low to moderate risk included women with a systematic coronary risk evaluation (SCORE) of <5%. High cardiovascular risk was defined as a 5-10% risk of fatal CVD within 10 years and included women with cholesterol levels >8 mmol/L, blood pressure ≥180/110mmHg, diabetes, or eGFR of 30-59 ml/min/1.73m2. Very high cardiovascular risk was defined as a ≥10% risk of fatal CVD within 10 years and included women with a history of CVD (as previously defined), diabetes in combination with current smoking, hypercholesterolemia or hypertension, diabetes with microalbuminuria, or eGFR of <30 ml/min/1.73m2. CVD included the presence of at least one of the following: heart failure diagnosis, coronary heart disease, angina, myocardial infarction, or stroke.

The Strengthening the Reporting of Observational Studies in Epidemiology (STROBE) guideline was applied to report this cross-sectional study, which complies with the Declaration of Helsinki. The study was approved by the institutional review board of the Centers for Disease Control and Prevention (Protocol #98-12 and Protocol #2005-06). Documented consent was obtained from participants.

Sample size calculation

We conservatively estimated the prevalence of CVD (including stroke, ischemic heart disease, and heart failure) to be 3.5% in women aged 20-55 without fibroids and 50-80% higher (e.g., 5%) in women with fibroids [7]. We thus calculated a minimum sample size of 3,370 to detect differences with alpha = 0.05 and 1 - beta = 0.80.

Statistical analyses

We aggregated the data across the four cycles, as previous research has shown similar fibroid prevalence rates across cycles [9]. We stratified the sample across the median age of the sample population and assessed the distribution and differences in baseline characteristics by fibroid status in the total group and by age, using appropriate statistical tests. We first calculated Kendall’s tau correlation coefficients for the presence of fibroids and well-known associated factors, such as ancestry, hypertension, BMI, diabetes, chronic kidney disease, and hypercholesterolemia. We then conducted univariable and multivariable logistic regression analyses with clinical and statistically relevant variables (at p<0.10) using forced entry to ensure that model assumptions were met. We also assessed crude and age-adjusted prevalence (using age-adjusted weights for the U.S. population) [17], as well as the multivariable association of very high cardiovascular risk with fibroids.

For the main outcome, we calculated crude and age-adjusted CVD prevalence by fibroid status and assessed potential predictors of CVD. We then assessed the independent association of CVD with fibroids. We used three adjustment models. Model A adjusted for age, BMI, ancestry, education level, smoking status, menopausal status, fasting total cholesterol, and presence of diabetes. Model B was further adjusted for systolic blood pressure. Model C was further adjusted for hemoglobin level. Finally, as a sensitivity analysis, we excluded women with a history of hysterectomy or bilateral oophorectomy from the main logistic regression analysis of an association between fibroids and CVD [10-12,18].

Model fit was assessed using the appropriate goodness-of-fit test for logistic regression models. Missing values were not imputed. We limited the use of p-values, expressing main outcomes as point estimates with 95% confidence intervals. A one-sided p-value was considered significant only with an a priori formulated direction of the outcome. Data were analyzed using IBM SPSS Statistics for Windows, Version 27.0 (Released 2020; IBM Corp., Armonk, New York, United States).

## Results

Fibroids

Of the initial sample of 6,508 women between 20 and 54 years old, 5,552 answered the question on fibroids, including 700 women with a history of uterine fibroids. Table 1 summarizes the sample characteristics by fibroid status and by age. Women with fibroids were older, had a higher education level, were more often of African ancestry and postmenopausal; had higher rates of hypertension, diabetes and CVD, and more frequently reported a history of hysterectomy or oophorectomy.

**Table 1:** Baseline characteristics of the participants *Data are mean (standard deviation) and our count (%) unless otherwise specified. Cardiovascular disease included a self-reported history of heart failure, coronary heart disease, angina, myocardial infarction, or stroke; Cardiovascular risk categories were based on the recommendation of the 2018 ESC/ESH Guidelines for the management of arterial hypertension. Low to moderate, high, or very high cardiovascular risk indicates respectively 5%, 5-10%, or ≥10% risk of fatal cardiovascular disease in 10 years. Variables with >5% missing values were cardiovascular risk (7%), hypertension treatment status (9%), BMI/obesity (10%), and the use of contraceptive hormones or HRT (36%). †No significant difference between fibroids and control (no fibroids). SBP: systolic blood pressure; DBP: diastolic blood pressure; CKD: chronic kidney disease; LDL: low-density lipoprotein; HDL: high-density lipoprotein; ESH: European Society of Hypertension; ESC: European Society of Cardiology

					All participants			20 to 35 y			>35 y		
Clinical Parameter*						Control 4852	Fibroids n=700		Control n=2771	Fibroids n=97		Control (n=2081)	Fibroids (n=603)
Age, years				34.6 (9.9)			43.6 (7.3)	27.1 (4.5)		30.3 (4.1)	44.5 (5.5)		45.8 (5.2)
Body mass index, kg/m^2^				28.6 (7.2)			30.5 (8.0)	28.0 (7.0)		31.9 (9.4)	29.5 (7.5)		30.3 (7.7)
Obesity, %				35.3			45.4	32.5		51.5	39.4		44.4
Hispanic, %				30.3			15.9	31.1		11.3	29.3		16.6
White-European, %				46.8			42.7	45.6		44.3†	48.2		42.5
African, %				18.2			38.0	18.2		38.1	18.2		38.0
High education level, %				52.3			61.6	54.7		53.6†	50.9		62.9
Ever smoked, %				37.3			42.6	32.9		37.1	43.2		43.5†
Pregnancies, N				3.0 (1.9)			3.2 (1.8)	2.6 (1.6)		3.3 (1.9)	3.5 (2.1)		3.2 (1.7)
			Life births, N	2.2 (1.4)			2.3 (1.3) †	1.8 (1.2)		2.2 (1.3)	2.6 (1.5)		2.3 (1.3)
Using sex hormones, %				25.1			28.8	28.5		15.2	21.1		31.0
Postmenopausal, %				10.5			28.6	1.7		9.3	28.0		31.7
Hysterectomy, %				5.6			39.6	0.4		10.3	12.3		44.3
Oophorectomy, %				4.8			29.0	1.2		12.4	9.6		31.7
		Bilateral		2.5			19.3	0.2		7.2	5.6		19.3
Hemoglobin, mg/dL				13.3 (1.3)			13.4 (1.3) †	13.1 (1.2)		13.3 (1.4) †	13.5 (1.3)		13.4 (1.3) †
SBP, mmHg				113.5 (14.7)			121.5 (18.5)	108.9 (10.3)		113.7 (15.0)	119.6 (17.3)		122.7 (18.7)
DBP, mmHg				68.1 (11.5)			74.2 (11.2)	64.4 (10.6)		68.0 (12.6)	73.2 (10.7)		75.2 (10.6)
Hypertension, %				15.3			33.4	2.4		14.1	24.6		35.7
Creatinine, micromole/L				60.1 (25.2)			65.4 (30.7)	57.6 (22.9)		58.1 (14.0)†	63.5 (7.6)		66.6 (32.4)
CKD, %				10.1			12.2	8.3		8.6†	12.3		12.7†
Glucose, mmol/L				4.9 (1.3)			5.4 (2.0)	4.7 (0.9)		4.8 (0.9) †	5.3 (1.7)		5.4 (2.1) †
Diabetes mellitus, %				4.8			10.0	1.9		4.8	7.2		10.7
Total cholesterol, mmol/L				5.2 (1.1)			5.2 (1.0) †	5.1 (1.2)		5.3 (1.0) †	5.2 (1.1)		5.2 (1.0) †
LDL-cholesterol				3.0 (0.9)			3.0 (0.8) †	2.9 (0.9)		3.2 (0.8) †	3.1 (0.9)		3.0 (0.8) †
HDL-cholesterol				1.5 (0.4)			1.5 (0.4) †	1.5 (0.4)		1.6 (0.4) †	1.5 (0.4)		1.5 (0.4) †
Hypercholesterolemia, %				17.6			21.9	17.1		21.5†	20.3		22.0†
Triglycerides				1.5 (1.0)			1.5 (1.5) †	1.4 (0.9)		1.3 (0.7) †	1.5 (1.1)		1.5 (1.6) †
Cardiovascular risk,%													
	Low to moderate			90.6			81.3	95.0		93.5†	85.0		79.3
	High			3.2			3.4†	2.9		3.3†	4.2		3.7†
	Very high			5.5			15.1	2.1		3.3†	10.8		17.0
Cardiovascular disease, %				2.5			7.3	0.9		0†	4.5		8.5

**Figure 1:**
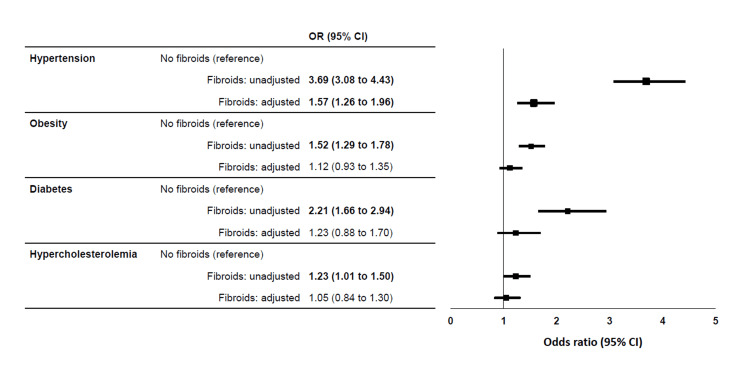
Odds ratio of cardiovascular risk factors in women with vs without fibroids Adjustment was for age, obesity, ancestry, diabetes, hypertension, hypercholesterolemia, high education level, smoking history, and menopausal status. Odds ratios (OR) with 95% confidence interval printed in boldface are statistically significant.

Table 2 shows correlations between risk factors and fibroids. In multivariable logistic regression analysis (excluding gynecological interventions), African ancestry was the main independent predictor of fibroids (odds ratio, 2.52; 95%CI, 1.96 to 3.23). In stratified analysis by ancestry, no difference was observed between women of African ancestry vs others in predictors of fibroids including age, BMI, education level, age at menarche, and smoking status (data not shown). Of the cardiovascular risk factors assessed, only hypertension was independently associated with fibroids (odds ratio, 1.57; 95%CI, 1.26 to 1.96) (Figure 1).

**Table 2:** Correlation analysis Variables that significantly correlate with fibroids and cardiovascular disease (at p</=0.10); Please see Methods and Materials section for definitions of hypertension and other aggregate clinical parameters. N.s.: not statistically significant

	Kendall's tau	
Clinical Parameter	Fibroids	CVD
Age	0.246	0.125
Fibroids	-	0.092
Hispanic vs other	-0.106	-0.032
African vs other	0.162	0.053
White vs Hispanic	0.076	n.s.
High education level	0.062	-0.034
History of hysterectomy	0.373	0.156
History of oophorectomy	0.300	0.134
Postmenopausal state	0.179	0.113
Age at menarche	-0.036	-0.030
Body mass index	0.066	0.060
Systolic blood pressure	0.132	0.077
Hypertension	0.202	0.168
Hypercholesterolemia	0.029	0.069
Hemoglobin	0.028	0.031
Ever smoked	0.036	0.058
Diabetes mellitus	0.076	0.119
Chronic kidney disease	0.023	0.077
Cardiovascular disease	0.092	-

Cardiovascular risk

**Figure 2:**
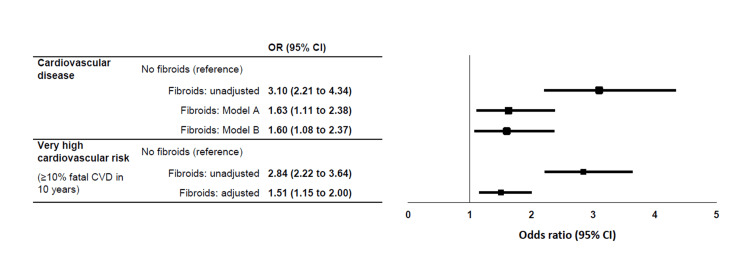
Odds ratio of cardiovascular disease and very high cardiovascular risk in women with vs without fibroids Cardiovascular disease (CVD) was based on being diagnosed with heart failure, coronary heart disease, angina, myocardial infarction, or stroke. Model A was adjusted for age, body mass index, ancestry, fasting plasma glucose and cholesterol, education level, smoking history, and menopausal status. Model B was further adjusted for systolic blood pressure. Very high cardiovascular risk was defined as a ≥10% risk of fatal CVD in 10 years (history of CVD, diabetes in combination with current smoking, hypercholesterolemia or hypertension; diabetes with microalbuminuria; or estimated glomerular filtration rate (eGFR) <30 ml/min/1.73m2). The adjusted model included age, body mass index, ancestry, education level, smoking history, and menopausal status

The crude prevalence of very high cardiovascular risk (≥10% fatal CVD in 10 years) was 15.1% in women with fibroids vs 5.5% in controls (respectively 11.0% vs 7.9% after age-adjustment; p=0.008), with an odds ratio in women with fibroids vs controls of 2.84 (95%CI, 2.22 to 3.64) and 1.51 (95%CI, 1.15 to 2.00) before and after adjustment for age, BMI, ancestry, education level, smoking history, and menopausal status, respectively (Figure 2).

Cardiovascular disease

Around 3% of participants (n=171) reported at least one cardiovascular event (2.2%, 2.9%, and 4.9% among those with Hispanic, White European, and African ancestry, respectively). Regarding the type of cardiovascular events reported, angina pectoris was most common (28% overall; 37%, 32%, and 18% for women of Hispanic, White-European, and African ancestry, respectively), followed by myocardial infarction (23% overall; 23%, 27%, and 20% for the respective ancestry groups), heart failure (22% overall; 23%, 16%, and 29% for the respective ancestry groups), and stroke (39% overall; 31%, 41%, 38% for the respective ancestry groups).

**Figure 3:**
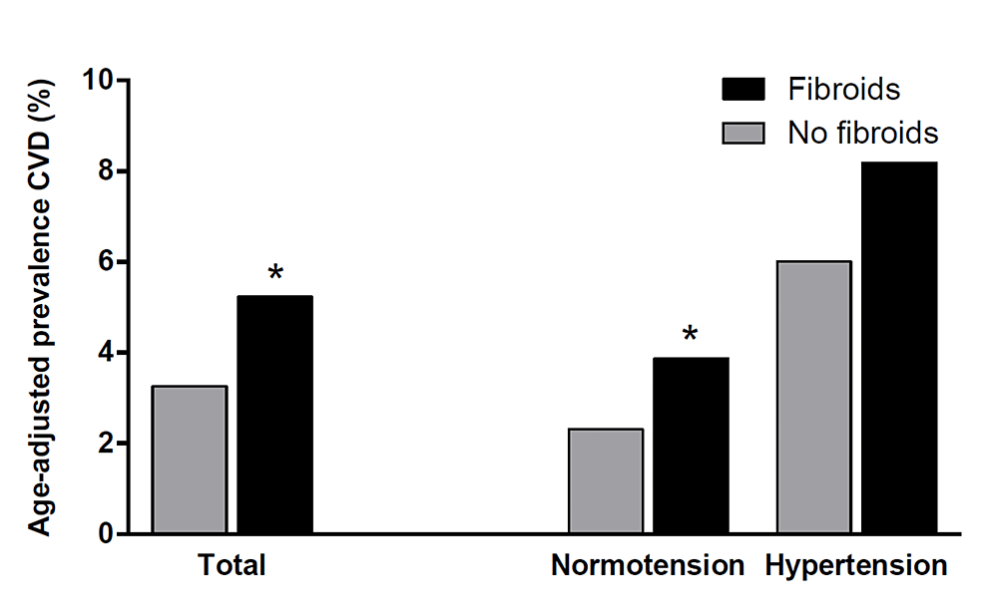
Age-adjusted prevalence of cardiovascular disease in women with and without fibroids by hypertension status *p <0.05 compared to women without fibroids CVD: cardiovascular disease

The prevalence of CVD in women with fibroids was 7.3% compared to 2.5% in the controls (p<0.001) (Table 1), with an age-adjusted CVD prevalence of 5.3% in women with fibroids vs 3.4% in controls (p=0.012), as shown in Figure 3. In univariable logistic regression, the odds ratio of CVD in women with fibroids was 3.10 (95% CI, 2.21 to 4.34), compared to controls (Figure 2). When stratified by age (at the population median), women with fibroids aged 35 years or younger had more hypertension, obesity, and diabetes, but not CVD, whereas women with fibroids aged 36 and older reported a higher prevalence of CVD, even when normotensive (Table 3).

**Table 3:** Cardiovascular risk and disease by age Fibroids and hypertension by age (population sample median age). Women with fibroids had greater CVD prevalence than women without fibroids even when normotensive. BMI: body mass index; CKD: chronic kidney disease; CVD: cardiovascular disease

	≤35 y			
Clinical Parameter	No Fibroids+ Normotension	Fibroids+ Normotension	No Fibroids+ Hypertension	Fibroids+ Hypertension
N	2596	79	65	13
African ancestry, %	17.3	32.9	41.5	53.8
BMI, kg/m^2^ (SD)	27.8 (6.8)	30.4 (7.5)	34.0 (9.0)	37.0 (12.1)
Diabetes mellitus, %	1.7	3.8	9.5	15.4
CKD, %	7.4	7.8	23.8	8.3
CVD, %	0.8	0	4.6	0
	35-54 y			
Clinical Parameter	No Fibroids+ Normotension	Fibroids+ Normotension	No Fibroids+ Hypertension	Fibroids+ Hypertension
N	1522	496	375	215
African ancestry, %	14.5	30.1	29.2	52.1
BMI, kg/m^2^ (SD)	28.1 (6.5)	28.8 (6.7)	33.1 (8.7)	32.7 (8.9)
Diabetes mellitus, %	8.5	4	16.1	21.9
CKD, %	8.5	6.4	22.2	22.3
CVD, %	2.7	6.1	9.9	12.6

Table 2 reports the parameters significantly correlated with the presence of CVD. Independent predictors of CVD were hysterectomy (odds ratio 2.50; 95%CI, 1.71 to 3.65), bilateral oophorectomy (2.42; 95%CI, 1.56 to 3.79), smoking history (1.79; 95%CI, 1.28 to 2.51), hypertension (1.66; 95%CI, 1.11 to 2.48), fibroids (1.52; 95%CI, 1.03 to 2.24), hypercholesterolemia (1.50; 95%CI, 1.02 to 2.22), age (1.07; 95%CI, 1.05 to 1.10), and BMI (1.04; 95%CI, 1.02 to 1.06), but not ancestry. After adjusting for age, BMI, ancestry, fasting plasma glucose and cholesterol, education level, smoking history, and postmenopausal status (Model A), the odds ratio of CVD with fibroids was 1.63; 95%CI, 1.11 to 2.38. After further adjustment for systolic blood pressure (Model B), the odds ratio became 1.60; 95%CI, 1.08 to 2.37 (Figure 2), with a similar outcome after adjustment for hypertension instead of systolic blood pressure (1.58; 95%CI, 1.07 to 2.33). Adding hemoglobin levels to the regression analysis (Model C) had no influence on the odds ratio (1.58; 95%CI, 1.07 to 2.35). After excluding women with a history of hysterectomy or bilateral oophorectomy the odds ratio for total CVD in women with fibroids remained statistically significant (Model B, odds ratio 1.77; 95%CI, 1.02 to 3.07).

## Discussion

We present data indicating that women with a self-reported history of fibroids have a greater prevalence of CVD, including ischemic heart disease, heart failure, and stroke. This contemporary explorative analysis fills a gap in knowledge of women’s health. Importantly, the data of this cross-sectional study suggest that the association between a history of fibroids and CVD is also present in women who did not have surgery for fibroids, and also in normotensive women with fibroids. Women with fibroids were older and more often of African ancestry than women without fibroids, but the association with CVD persisted after adjustment for age, BMI, ancestry, and systolic blood pressure. The findings suggest that fibroids are associated with systemic perturbations that increase cardiovascular risk, rather than being a local uterine disease.

Our previous work has shown that fibroids are associated with hypercontractility of resistance arteries and with hypertension [5,7,8]. However, in the current analysis, women with fibroids and normotension also had higher cardiovascular risk than controls. The nature of this association remains largely unclear. Women with fibroids are incompletely phenotyped for cardiovascular and blood pressure characteristics; therefore, we cannot exclude that women classified as normotensive may have had high mean 24-hour or nocturnal blood pressure readings, or, alternatively, that blood pressure levels associated with CVD are lower for women in general, or with fibroids. Fibroids also have been associated with obesity, diabetes, and hypercholesterolemia [5-7], though the findings are inconsistent. Nevertheless, these conditions likely contribute to the elevated cardiovascular risk associated with this condition. Previous studies have found CVD to be associated with hysterectomy and oophorectomy [10-12,18], but no such association with fibroids has been established.

We previously reported a higher prevalence of asymptomatic hypertension-mediated organ damage (with criteria including pulse pressure, aortic pulse wave velocity, ankle‐brachial index, electrocardiographic left ventricular hypertrophy, eGFR 30-60 mL/min/1.73 m2, or proteinuria) in young women with fibroids, compared to controls, of 66.7% versus 42.9% (p<0.001), respectively [7,16]. We attributed this finding to higher pulse pressure and pulse wave velocity in women with fibroids [7]. This finding aligns with our proposal of a common pathology of the vessel wall and fibroid smooth muscle [5], which may be associated with multiple factors, including genetic or epigenetic characteristics, the abundantly present ATP-generating enzyme creatine kinase, renin-angiotensin system activation, inflammation, environmental toxins, or some other cause [5-8,19-21].

The main strength of this study is that we address the risk of cardiovascular events in a female-specific condition, fibroids. CVD remains a major cause of premature death in women, and the association with commonly occurring fibroids is under-recognized and understudied. The greater occurrence of fibroids in women of African ancestry highlights the relevance of ethnic disparities in health and health equity. Though we adjusted the analysis for commonly known risk factors, the causal pathway of the association of fibroids and CVD is not known, so to avoid overadjustment, we reduced the precision of our statistical models. Therefore, we also present crude, unadjusted data.

Limitations of this study are the data collection using a large, cross-sectional population study containing historical data that we analyzed retrospectively. The most recent NHANES data asking questions about fibroids is from 2006. This area of women’s health is underfunded, despite the frequent occurrence of uterine fibroids [7,9,19-21]. Thus, more recent studies and data were not available to us. Symptomatic fibroids are reported in 12-14% of adult women [9], and these numbers likely underestimate the true disease burden due to asymptomatic or paucisymptomatic fibroids. More population-based epidemiological studies are needed to assess relevant trends in prevalence, disease burden, and CVD in relation to fibroids to advance the field of women’s health.

Despite the use of cross-sectional public health data, the presented analyses align with other work showing that women with symptomatic fibroids have higher cardiovascular risk [5-7,9-12,18]. Clustering of fibroids with cardiovascular events may require early management. Yet, awareness of this association in the scientific community is low, and fibroids are rarely mentioned as a potential cardiovascular risk factor [1,2,4,16,19-22]. Hypothesis-generating data may help draw attention to the potential systemic cardiovascular implication of this female-specific risk factor. The presented data could be useful to design dedicated, prospective studies to better phenotype women with fibroids and their clinical and pathological cardiovascular characteristics. To our knowledge, no screening program includes this condition, so we cannot exclude surveillance bias in the data. However, in our previous study, women who underwent surgery for fibroids had significantly more hypertension than women who underwent other gynecological surgery [5]. In addition, in the absence of screening, we cannot know the cardiovascular risk of asymptomatic fibroids. This lack of data should be a subject of further study.

## Conclusions

We observed an independent association between prevalent CVD and fibroids in normotensive as well as hypertensive women in the general population, compared to controls. As fibroids occur with greater frequency and severity in women of African ancestry, it is important to consider how gender, sex, ancestry, socio-economic deprivation, environmental stressors, and unequal access to healthcare might reinforce existing health disparities in the treatment of this understudied condition and its association with CVD. The presented data highlight the relevance of a female-specific research agenda, particularly regarding underserved populations that experience health inequities. The data could be used to design preventive strategies to reduce the cardiovascular risk associated with uterine fibroids and to better phenotype women with fibroids with regard to cardiovascular events.
